# Correlational and Regression-Based Insights into Shoulder and Core Stability Tests in Amateur Athletes: Reference Values and Sex-Based Comparison

**DOI:** 10.12669/pjms.42.3.13281

**Published:** 2026-03

**Authors:** Nazar Deen, Saeed Akhter, Salman Khan, Sadia Akhter

**Affiliations:** 1 Nazar Deen, MSAPT, Institute of Physical Therapy & Rehabilitation, Jinnah Sindh Medical University, Karachi, Pakistan; 2 Saeed Akhter, PhD, Institute of Physical Therapy & Rehabilitation, Jinnah Sindh Medical University, Karachi, Pakistan, Sindh Institute of Physical Medicine & Rehabilitation, Chand Bibi Road, Karachi, Pakistan; 3 Salman Khan, MSAPT, Institute of Physical Therapy & Rehabilitation, Jinnah Sindh Medical University, Karachi, Pakistan, Sindh Institute of Physical Medicine & Rehabilitation, Chand Bibi Road, Karachi, Pakistan; 4 Sadia Akhter, MSAPT*, Institute of Physical Therapy & Rehabilitation, Jinnah Sindh Medical University, Karachi, Pakistan

**Keywords:** Core performance test, Normative values, Prediction, Shoulder, Stability

## Abstract

**Objective:**

To investigate correlation of core and shoulder physical tests, prediction of interdependent relationship and to develop reference values.

**Methodology:**

The study was conducted at Jinnah Sindh Medical University (JSMU), on amateur athletes: (n=70), recruited from JSMU sport society. Data were analyzed using IBM-SPSS version 23.0. Side bridge test right and left (SBTR and SBTL), flexor endurance test (FET), closed kinetic chain upper extremity stability test male/female (CKCUEST-M/F) outcomes and scores of CKCUEST were analyzed with gender using independent sample t test, reference values were established. Simple and multiple linear regression of normalized CKCUEST score was analyzed with independent variables.

**Results:**

Male had significantly higher mean values on SBTR and SBTL (p<0.01*) while no significance for FET, and CKCUEST outcomes. Male had significantly higher Core Endurance Asymmetry (CEA) (p=0.024*). ER/IR strength did gives 25.1% negative correlation with CEA, whereas Normalize scores of CKC-T did give 27.9% positive correlation with CEA and 50.6% positive correlation with power scores of CKCUEST. Simple and multiple linear regression models revealed that a one unit increase in Power score of CKCUEST and CEA will give significant increase in Normalize score of CKCUEST for samples. R-square of multiple regression model showed 30% variation in Normalize score of CKCUEST was explained by the studied predictors.

**Conclusion:**

Gender alone may not determine stability and ER/IR strength ratios seems less predictive comparing core engagement and coordination for stability. CEA and power emerge as stronger predictors. CEA prevails in male.

## INTRODUCTION

Overhead activities at certain speeds needs coordination, flexibility, strength, synchronicity and motor control at shoulder level which in turn requires strength and endurance at core as well.^1^ Core stability provides foundation and have an active role in upper limb activities.^2^ Literature supports established link between core stability and athletic performance^3^, having strong positive correlation.^4^ Similarly, high core strength is associated with greater shoulder stability and performance.^5^ Age has no effect but gender matters, female exhibiting greater extensor trunk endurance while male have greater lateral core endurance.^6^ The CKCUEST primarily reflects the dynamic stability of the glenohumeral joint.^7^

Thoracic mobility, and trunk endurance are closely linked to CKCUEST performance, highlighting its association with core function in overhead athletes.^7^ Men demonstrated greater lower limb strength, power, and trunk endurance measured by the plank and side plank tests compared to women, these tests have been previously validated as reliable tools for assessing trunk muscle endurance.^8^ The CKCUET test is a low cost and valid functional performance test for shoulder and having good to excellent reliability.^9^

Okada et al., 2011 at Indiana state university observed that certain core stabilizers activate consistently before limb movements. Their findings emphasize the role of kinetic chain integrated activity in initiating efficient whole-body movement.^10^ Athletes who later experienced trunk injuries showed weaker and more asymmetrical core stability than those who remained uninjured.^11^

However, such relationship has not been evaluated among the stability tests of the two region which operates in integrated kinetic chain. To the best of our knowledge no such study has been done to corelate core tests and to predict certain correlation, thus the aim of present study is to establish the reference values, to corelate and predict the interlinked dependency of one on the other; furthermore, either core asymmetries affect functional performance outcome of the athletes.

## METHODOLOGY

This study was conducted from April 2024 to May 2025 in physiotherapy laboratory setting at JSMU, where amateur athletes were analyzed to establish as first novel reference values generated research.

### Ethical approval:

The institutional review board (IRB) of JSMU approved the study with the registration number JSMU/IRB/2024/842, dated March 11, 2024. Informed consent was provided to each participant and permission were obtained from relevant department.

### Inclusion criteria:

 Athletes’ age between 18 to 30 years, both gender, with full functional ranges at shoulder and lumbar region, involved in sports like cricket, volleyball, tennis players, badminton, rackets and any other seasonal sports at university level, were recruited.

### Exclusion criteria:

- Active pathologies, trauma, cardiorespiratory complaints, neuromuscular problem, any surgery at cervical, shoulder and lumbar region and any other miscellaneous complaints hindering study protocols were excluded.

### Sample size estimation:

Confidence interval 95%, relative correlation between upper-extremity performance and UQYBT Score is^12^ 0.46 with power 80% power (β=0.2), then the estimated sample size was n=35, for generalizability purpose it was increased to 70.^3^

### Recruitment of participant:

The JSMU sport society was updated and informed consent, study protocols were sent to them and all amateur athletes were recruited, using the convenience sampling method. Experienced physiotherapist collected their baseline sociodemographic information including age, sex, BMI and another predesigned questionnaire with question about type of sport, frequency of playing and handedness were noted. In second phase the selected athletes were brought to the physical performance test session including: FET, SBTR, SBTL and CKCUEST M and F version were incorporated. Proper warm up for three minutes followed by three minutes rest and intersession rest of five minutes were assured. Each protocol was done for single and noted in a predesigned chart.

### Study variables:

- FET was used to observe the anterior lumbar core endurance. Participant sat on a bench with hips and knees at 90 degrees, arms folded across chest. The therapist started the timer and observed for test protocols and second one moved the wooden board back 10cm, the client held a 60-degree angle. The termination criteria were set as significant change in trunk position occurred (deviation from neutral spine or increased low-back arch), shoulders went rounded forward or the back touched the backrest and inability to maintain the 60 degree position. Time noted in seconds, for single measurement.
- SBT for both sides were conducted to check for lateral core endurance. SBT involves a static hold side-lying position supported by an elbow and feet lifting the hips and torso to form a straight line and holding the position for as long as possible.
- CKCUEST, participant’s hands were apart by 36 inches or 91.44cm distance marked by tape. The assessor was instructing the participants to touch their left hand with their right hand and then their right hand with their left hand for 15 seconds. During the procedure, the upper extremities were perpendicular to the ground. Single trial of 15 seconds was done. A therapist was verbally indicating the start and end of tests, while other one was counting touches. The CKCUEST was providing three scores: (1) Number of touches score; (2) Normalized score = Average number of lines touched /Height (inch.) and (3) Power score = 68% Weight × Average number of lines touched / 15 7.
- IR/ER strength was measured in Newton by a myometer.

### Statistical analysis:

Data analyzed using IBM-SPSS version 23.0; Means with standard deviations (SD) descriptive on SBT, FET and CKCUEST were also reported. Normalize Scores of CKCUEST, its power, ER/IR strength and CEA presented with mean and SD. Mean of all these parameters were compared between male and female samples using independent sample t-test. Spearman Rank correlation was reported to study the correlation analysis among variables, simple and multiple regression models were developed to predict the Normalize score of CKCUEST using Power scores of CKCUEST, ER/IR strength and CEA.

## RESULTS

Male athletes have significantly high IR and ER isometric strength; IR strength is greater than ER in both genders. SBT of both sides showing two fold longer time than female athletes indicating better lateral core endurance. Table-I FET, no significant relation exists although male hold more on average and 14 seconds difference is notable and directing borderline significance. Similarly, in CKCUEST show equal performance p-value 0.59. Table-II shows no statistical significance. Female showed marginally high normalized CKCUEST score while male exhibit high power score but both comparisons were insignificant and same reflection noted for IR/ER ration across gender. In CEA, significance was noted (p-value = 0.024*), pointing that bilateral core training and correction strategies would be better and reduces susceptibility of an athlete to injury.

**Table-I:** Mean Comparison of Side Bridge, Flexor Endurance, CKCUEST outcomes and IR/ER strength with Gender.

Parameters	Male (n=40)		Females (n=30)		p-value
	Mean	SD	Mean	SD	
SBTR	41.7	19.2	21.2	13.6	<0.01*
SBTL	44.1	20.8	24.2	15.0	<0.01*
FET in seconds	60.7	32.2	46.8	26.6	0.059
CKCUEST	16.1	3.4	16.6	3.7	0.59
IR (dominant side) in Newton	57.6	18.3	36.1	7.9	<0.01*
ER (dominant side) in Newton	56.6	17.1	34.0	11.1	<0.01*

**Table-II:** Mean Comparison of Normalize Scores of CKCUEST, ER/IR strength and CEA with Gender.

Normalize score of CKCUEST	0.24	0.05	0.26	0.06	0.16
Power score of CKCUEST	44.32	15.39	42.25	13.40	0.55
ER/IR Strength Ratio	1.78	.50	1.98	0.73	0.16
Core Endurance Asymmetry	9.55	8.13	5.40	6.37	0.024*

* p<0.05 was considered Statistically Significant using Independent sample t-test.

Table-III shows Inverse relationship between the ER/IR and CEA. CEA showed a positive correlation with the normalized CKCUEST score, whereas power and normalized scores of CKCUEST showed 50.6% correlation. In Table-IV Both regression models revealed that a one unit increase in Power score of CKCUEST and CEA will give significant increase in Normalize score of CKCUEST for samples considered statistically significant (p<0.05), R-square of multiple regression model showed 30% variation in Normalize score of CKCUEST was explained by the studied predictors.

**Table-III:** Spearman Rank Correlation analysis of ER/IR strength, CEA and CKCUEST Scores.

Parameters		ER/IR Strength Ratio	CEA	Normalize score of CKCUEST	Power score of CKCUEST
ER/IR Strength Ratio	r-value	1.000			
	p-value	-			
CEA	r-value	-0.251	1.000		
	p-value	0.036*	-		
Normalize score of CKCUEST	r-value	-0.022	0.279	1.000	
	p-value	0.85	0.019*	-	
Power score of CKCUEST	r-value	-0.026	0.141	0.506	1.000
	p-value	0.831	0.243	<0.01*	-

* p<0.05 was considered statistically significant.

**Table-IV:** Prediction of Normalized CKCUEST scores with Power Score CKCUEST, ER/IR strength and CEA.

Independent Variables	Simple Linear Regression β (95% C.I)	Multiple Linear Regression β (95% C.I)
Power score of CKCUEST	0.002* (0.001-0.003)	0.002* (0.001-0.003)
ER/IR Strength Ratio	-0.003 (-0.025-0.019)	0.003 (-0.016-0.021)
CEA	0.001* (0-0.003)	0.002* (0-0.003)

Dependent variable: Normalize score of CKCUEST;

* β was considered statistically significant with p<0.05; Adjusted R-Square for Multiple Linear Regression was 30%.

**Fig.1:**
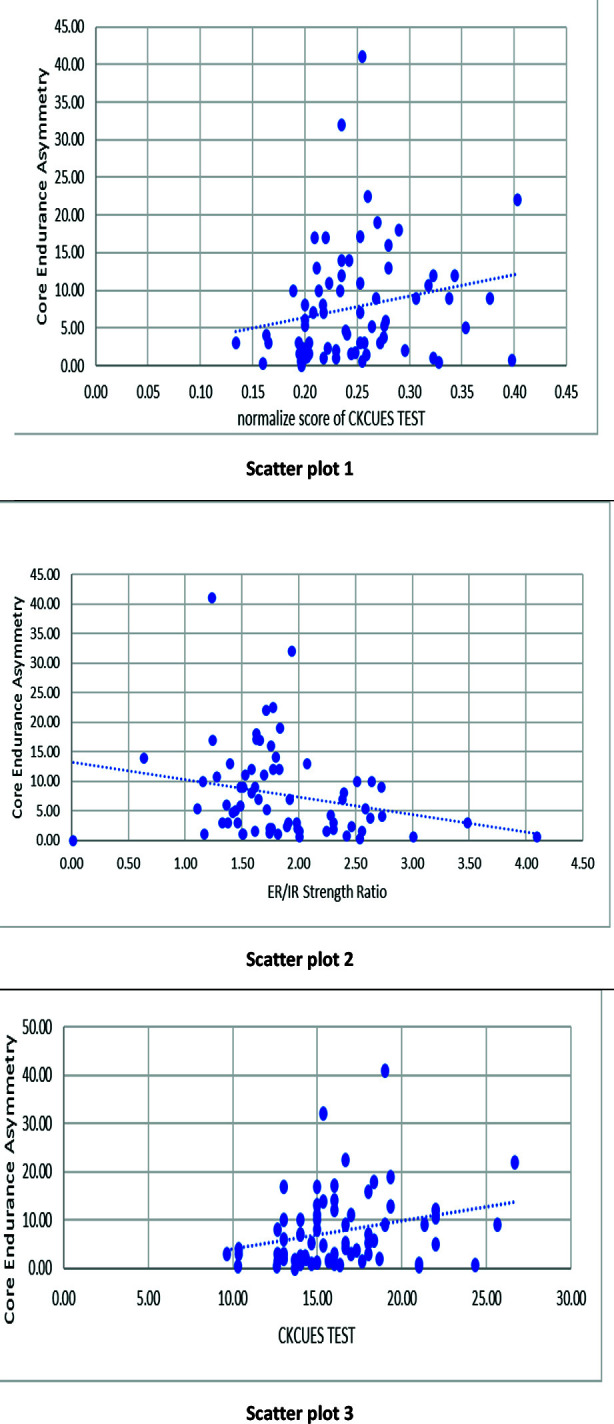
Scatter plot 1 showing positive linear relationship R-square showed 4.2% variation, plot 2 showing negative linear relationship-square showed 5.6% variation. Plot 3 showing positive linear relationship and 6.9% variation.

Fig.1 illustrates scatter plots, Scatter plot one showing positive linear relationship R-square showed 4.2% variation, plot two showing negative linear relationship-square showed 5.6% variation. plot three showing positive linear relationship and 6.9% variation.

## DISCUSSION

This study investigated reference values of FET, SBTR, SBTL, CKCUEST (M/F). Findings suggest that CEA is present between gender. ER/IR ratio is not but Power score and CEA are significant predictors. The lumbar core provides stable platform to distal muscles to operates and transfers considerable force to them in a substantial kinetic chain; the shoulder core works with later core supports.^13^ In both SBT, male outperformed female (p-value <0.01*), the nearly 2 × longer hold time suggests meaningful gender differences, possibly due to greater muscle mass and stability in males, The asymmetry in results across both sides supports stronger bilateral core endurance in males. Hence the holding time in our participants is below the findings of a study; which have 60 seconds for right and 62 seconds for left SBT, although their study’s SBTL has raised value as this study had.^14^ Exceptionally, another study had raised values for lateral endurance of both sides; (101.90±47.99; dominant and 97.64±49.94; non-dominant side), almost two-fold comparing this study, this difference may be due amateur participant and socio-cultural context in our research.^15^ Additionally, our male participants have raised values and asymmetry in both SBTs while above studies did not elaborate such gender difference.

In FET, males showed higher hold, but the difference just missed statistical significance (p = 0.059). Given the moderate-to-high variability (SD ~30 seconds), the sample may not have had enough power. Clinically, the 14 seconds (Table-I) difference is still notable. Observations of de Oliveira IO et al., are almost consistent with us except FET (154.19± 44.21).^16^ In this study male has outperformed female in all tests. One study suggests 178 seconds,^14^ for FET which is much high scores than our value, probably due to early mentioned reason of amateur athletes, our FET values are not consistent with other studies. Conversely the findings of a current study on male elite athletes are highly consistent; their scores for SBTR were 50.4 ± 20.5 seconds and for SBTL was 48.2 ± 19.3 seconds, similarly for FET test 81.8 ± 32.7 seconds which are in nearer range to our observations.^5^

In CKCUEST, athletes performed same in terms of repetitions as no significant difference between genders. A study by Tucci HT et al, in which male/female participant with parameters of: Sedentary; Active; and sub impingement syndrome (SIS) were studied and concluded that test reference score were less than reference scores (male 18.5/ female 20.5,considering number of touches: female > male in above three parameters.^17^ In our study female have relatively more touches than male although insignificant, whereas our CKCUEST touches lies in between scores of their sedentary and SIS participant’s touches and almost in nearer ranges to the standard reference scores. In a recent study on female athletes (15.77 ± 3.91 touches), are highly consistent to us.^18^ CKCUEST normalized and power scores (~42–44 W) of this study are modest, showing relative lower stability linking to injury probabilities are in nearer range to findings of Teixeira AL et al.^19^ while power scores are far below to most international references.^17^

Further by analyzing the CEA, a significant difference is observed; males show significantly greater CEA consistent with Maly T et al., they studied asymmetries in detail and concluded that significant differences are there in soccer players and highest is present between dominant and non-dominant in postural stability, further proposed that constant loading of one side of body for long leads to strength and balance asymmetry.^20^ Literature on asymmetries is daunting and contentious.^21^ Bailey et al. investigated that force asymmetries during bilateral tasks and do report a negative association between asymmetry and maximal strength performance.^22^

Similarly, our correlation analysis of studied parameters; upper extremity ER/IR strength did gives 25.1% negative correlation (Correlation (r = -0.251, p = 0.036*) with CEA, whereas Normalize scores of CKCUEST did give 27.9% positive correlation with core endurance asymmetry and 50.6% positive correlation with power scores of CKCUEST. All these correlations were found statistically significant with p<0.05.Considering the simple linear and multiple regression of the CKCUEST, no such studies has been identified for comparison anyhow a significant correlation as both simple and multiple linear regression models revealed that a one unit increase in Power score of CKCUEST and CEA (Indicates that increased CEA is associated with higher CKCUEST scores, Effect is small but real) will give significant increase in Normalize score of CKCUEST for samples considered statistically significant (p<0.05) while IR/ER ratio does not meaningfully predicting normalized CKCUEST score. Schilling DT and Elazzazi AM, 2021 revealed inverse correlation between CKCUEST power and IR/ER isometric strength.^23^ Whereas, coordination between arm and eye improves perception and synergism, further potentiates the motor skills in participation.^24^

### Strength:

This is the first novel study in Pakistan addressing the references values of stability tests in athletes, as they are quite often prone to musculoskeletal injuries due to multifactorial reason, such study will guide for better training regimen. It complies with international studies and providing a thorough impression of current values and performance status.

### Limitations:

It includes small sample size and single center study (selection biasness), for the mentioned reasons and low resources the references values were established rather than normative. Further in-depth and multicenter, large sports club research conduction with large sample size would provide better normative reflections and generalizability of the findings.

## CONCLUSION

Male are more asymmetrical in core endurance; shoulder strength is less predictable than core asymmetry and power, may not directly influence closed kinetic chain upper limb stability, reference value are below to normative values. Large sample size required for better outcomes.

### Authors Contribution:

**ND:** Idea generation, study design, data collection manuscript writing, and responsible for the accuracy of the study.

**SA:** Step by step review, study design, English language correction.

**SK** and **SAR:** Data collection, methodology.

All authors have approved the final version of the manuscript.

### Abbreviations:

**JSMU:** Jinnah Sindh Medical University.

**CEA:** Core Endurance Asymmetry.

**CKCUES M/F:** Closed Kinetic Chain Upper Extremity Stability Test Male/Female.

**SBTR:** Side Bridge Test Right.

**SBTL:** Side Bridge Test Left.

**FET:** Flexor Endurance Test.

**ER/IR:** External Rotation/Internal Rotation.
